# Germline *ATM* variants predispose to melanoma: a joint analysis across the GenoMEL and MelaNostrum consortia

**DOI:** 10.1038/s41436-021-01240-8

**Published:** 2021-07-14

**Authors:** B. Dalmasso, L. Pastorino, V. Nathan, N. N. Shah, J. M. Palmer, M. Howlie, P. A. Johansson, N. D. Freedman, B. D. Carter, L. Beane-Freeman, B. Hicks, A. Molven, H. Helgadottir, A. Sankar, H. Tsao, A. J. Stratigos, P. Helsing, R. Van Doorn, N. A. Gruis, M. Visser, K. A. W. Wadt, G. Mann, E. A. Holland, E. Nagore, M. Potrony, S. Puig, C. Menin, K. Peris, M. C. Fargnoli, D. Calista, N. Soufir, M. Harland, T. Bishop, P. A. Kanetsky, D. E. Elder, V. Andreotti, I. Vanni, W. Bruno, V. Höiom, M. A. Tucker, X. R. Yang, P. A. Andresen, D. J. Adams, M. T. Landi, N. K. Hayward, A. M. Goldstein, P. Ghiorzo, The GenoMEL, MelaNostrum consortia

**Affiliations:** 1 IRCCS Ospedale Policlinico San Martino, Genetics of Rare Cancers, Genoa, Italy; 2 Department of Internal Medicine and Medical Specialties, University of Genoa, Genoa, Italy; 3 Oncogenomics Laboratory, QIMR Berghofer Medical Research Institute, Brisbane, QLD Australia; 4 Division of Cancer Epidemiology and Genetics, National Cancer Institute, Bethesda, MD USA; 5 American Cancer Society, Atlanta, GA USA; 6 Cancer Genomics Research Laboratory, Leidos Biomedical Research, Frederick National Laboratory for Cancer Research, Frederick, MD USA; 7 Gade Laboratory for Pathology, Department of Clinical Medicine, University of Bergen, Bergen, Norway; 8 Department of Pathology, Haukeland University Hospital, Bergen, Norway; 9 Department of Oncology Pathology, Karolinska Institutet and Karolinska University Hospital Solna, Stockholm, Sweden; 10 Wellcome Sanger Institute, Wellcome Genome Campus, Hinxton, Cambridge, UK; 11 Wellman Center for Photomedicine, Department of Dermatology, MGH Cancer Center, Massachusetts General Hospital, Boston, MA USA; 12 First Department of Dermatology–Venereology, Andreas Sygros Hospital, Medical School, National and Kapodistrian University of Athens, Athens, Greece; 13 Department of Dermatology, Oslo University Hospital, Oslo, Norway; 14 Department Dermatology, Leiden University Medical Center, Leiden, The Netherlands; 15 Department of Clinical Genetics, University Hospital of Copenhagen, Copenhagen, Denmark; 16 Centre for Cancer Research, Westmead Institute for Medical Research, University of Sydney, Westmead, Australia; 17 Department of Dermatology, Instituto Valenciano de Oncologia, Valencia, Spain; 18 Biochemistry and Molecular Genetics Department, Melanoma Unit, Hospital Clínic de Barcelona, IDIBAPS, Universitat de Barcelona, Barcelona, Spain; 19 Centro de Investigación Biomédica en Red de Enfermedades Raras (CIBERER), Barcelona, Spain; 20 Dermatology Department, Melanoma Unit, HospitalClínic de Barcelona, IDIBAPS, Universitat de Barcelona, Barcelona, Spain; 21 Immunology and Molecular Oncology Unit, Veneto Institute of Oncology IOV-IRCCS, Padua, Italy; 22 Institute of Dermatology, Catholic University of the Sacred Heart, Rome, Italy; 23 Fondazione Policlinico Universitario A. Gemelli, IRCCS, Rome, Italy; 24 Dermatology, Department of Biotechnological and Applied Clinical Sciences, University of L’Aquila, L’Aquila, Italy; 25 Dermatology Unit, Maurizio Bufalini Hospital, Cesena, Italy; 26 Dépatement de Génétique Moléculaire, Hôpital Bichat-Claude Bernard, Paris, France; 27 Section of Epidemiology and Biostatistics, Leeds Institute of Cancer and Pathology, University of Leeds, Leeds, UK; 28 Department of Cancer Epidemiology, H. Lee Moffitt Cancer Center and Research Institute, Tampa, FL USA; 29 Department of Pathology, Oslo University Hospital, Oslo, Norway; 30 Divison of Cancer Epidemiology and Genetics, National Cancer Institute, National Institutes of Health, Bethesda, MD USA

## Abstract

**Purpose:**

Ataxia–Telangiectasia Mutated (*ATM*) has been implicated in the risk of several cancers, but establishing a causal relationship is often challenging. Although *ATM* single-nucleotide polymorphisms have been linked to melanoma, few functional alleles have been identified. Therefore, *ATM* impact on melanoma predisposition is unclear.

**Methods:**

From 22 American, Australian, and European sites, we collected 2,104 familial, multiple primary (MPM), and sporadic melanoma cases who underwent *ATM* genotyping via panel, exome, or genome sequencing, and compared the allele frequency (AF) of selected *ATM* variants classified as loss-of-function (LOF) and variants of uncertain significance (VUS) between this cohort and the gnomAD non-Finnish European (NFE) data set.

**Results:**

LOF variants were more represented in our study cohort than in gnomAD NFE, both in all (AF = 0.005 and 0.002, OR = 2.6, 95% CI = 1.56–4.11, *p* < 0.01), and familial + MPM cases (AF = 0.0054 and 0.002, OR = 2.97, *p* < 0.01). Similarly, VUS were enriched in all (AF = 0.046 and 0.033, OR = 1.41, 95% CI = 1.6–5.09, *p* < 0.01) and familial + MPM cases (AF = 0.053 and 0.033, OR = 1.63, *p* < 0.01). In a case–control comparison of two centers that provided 1,446 controls, LOF and VUS were enriched in familial + MPM cases (*p* = 0.027, *p* = 0.018).

**Conclusion:**

This study, describing the largest multicenter melanoma cohort investigated for *ATM* germline variants, supports the role of *ATM* as a melanoma predisposition gene, with LOF variants suggesting a moderate-risk.

## INTRODUCTION

Melanoma is the deadliest form of skin cancer, with a worldwide increasing incidence and burden, especially in countries with a majority of light-skinned individuals and a high ultraviolet (UV) radiation index/exposure [1]. The etiology of melanoma is complex, resulting from the interplay of environmental, host, and hereditary factors.

Approximately 5–12% of melanomas occur in individuals with a family history of melanoma or in cancer syndrome families. However, germline variants in known high-risk predisposition genes indicate heritability in less than half of the patients, frequently attributable to pathogenic variants in *CDKN2A* and, much less frequently, in *CDK4*. Additional genes, yet to be discovered, may also contribute to melanoma susceptibility, as has been shown for pathogenic variants of *POT1*, *BAP1*, *TERT*, *ACD*, and *TERF2IP* associated with less than 10% of melanomas accumulated within families [2]. More recently, other susceptibility genes such as *GOLM1*, *EBF3*, *POLE*, and *NEK11* have been associated with melanoma risk but not sufficiently validated [3]. Indeed, much of the missing heritability for melanoma may be due to the inheritance of multiple low-to-moderate risk alleles and/or shared environmental exposures that predispose to melanoma, culminating in a familial pattern of melanoma inheritance [4]. For example, several variants in *MC1R*, as well as *MITF* p.Glu318Lys, act as low/moderate risk variants for melanoma [2]. Moreover, pathogenic variants in novel candidate genes with possible moderate risk, such as those causing oculocutaneous albinism, have been reported in melanoma families, but larger studies are needed to fully elucidate their role [5].

Melanoma can also be a subordinate cancer in the context of other multitumor cancer syndromes for which heritability is underlined by a combination of high and moderate-risk genes [4]. Some of these moderate-risk genes predispose to different types of cancers. Variant interpretation in genes with likely moderate-low risk is particularly complex because of incomplete cosegregation with the disease and incomplete penetrance. Therefore, missense substitutions in these genes are often classified as variants of uncertain significance (VUS), lacking clearly defined risk estimates.

Germline pathogenic variants in the ataxia–telangiectasia mutated (*ATM*) gene predispose to multiple cancers. *ATM* is a large (66 exon) gene [6] and has, therefore, a high number of nucleotide substitutions for primarily stochastic reasons. The ATM protein is a serine/threonine kinase involved in the DNA damage response. In particular, ATM is activated upon DNA double-strand breaks caused by ionizing radiation, oxidative stress (ROS) and, indirectly, and by DNA damage caused by UV radiation, among other functions [6, 7]. Indeed, *ATM*-null cells have a high chromosomal aberrations rate and show a lack of DNA damage repair following exposure to ionizing radiation [8].

Biallelic *ATM* loss-of-function (LOF) variants result in ataxia–telangiectasia (AT), also known as Louis–Barr syndrome, an autosomal recessive disorder characterized by progressive cerebellar degeneration, ocular telangiectasias, immunodeficiency, and radiosensitivity, as well as predisposition to several hematologic and solid cancers. Similarly, heterozygotes for *ATM* variants have an increased risk of several malignancies. For example, *ATM* is an established breast cancer and pancreatic cancer predisposition gene and pathogenic variants in *ATM* have also been implicated in susceptibility to gastric cancer and prostate cancer, suggesting that the *ATM* tumor spectrum is likely broad [6].

Recent studies, including the largest meta-analysis of melanoma genome-wide association studies (GWAS) to date, have linked specific low-risk variants in *ATM* with melanoma, although the functional alleles have not yet been determined [9]. In addition, we recently found *ATM* LOF or potentially deleterious variants in up to 3% of high-risk melanoma patients [10, 11]. Therefore, we conducted a multicenter study among the GenoMEL (https://genomel.org/) and MelaNostrum (https://dceg.cancer.gov/research/cancer-types/melanoma/melanostrum/members) melanoma genetics consortia to further investigate the impact of *ATM* on the risk of developing melanoma.

## MATERIALS AND METHODS

### Study data sets

From centers across 22 sites of the GenoMel and MelaNostrum consortia in Europe, the United States, and Australia, we retrospectively collected information on the germline *ATM* status of 2,104 unrelated cutaneous melanoma cases, who were either probands from melanoma-prone families, multiple primary melanoma (MPM) cases, or melanoma cases belonging to case–control or cohort studies who tested negative for *CDKN2A* and *CDK4* (Table 1 and Supplementary Methods). *ATM* genotyping was performed via panel, exome, or genome sequencing (ES/GS) at each recruiting center. In addition to *ATM* germline variants, each participating group was asked to provide clinical information on melanoma cases included in the study: sex, age, age at diagnosis, and personal and family history of nonmelanoma cancers. Availability of clinical information varied by contributing center. Groups also provided information on cosegregation of *ATM* variants in cases from melanoma-prone families, if available.

**Table 1:** Study data set from the ten participating groups.

Participant group^a^	Sporadic cases from case–control cohorts	Sequencing method	MPM cases	Sequencing method	Familial melanoma probands	Sequencing method
NCI–USA	783/11	Panel/ES	3	ES	72	ES
OUH–Norway			279	ES		
UNIGE–Italy	22	Panel	84	Panel	167	Panel
QIMR Berghofer–Australia			3	ES	206	ES
MelaNostrum/NCI-USA					201	ES
Sanger–UK					90/25	ES/GS
KI–Sweden			10	Panel	73	ES
H. Bichat–France			20/11	ES/panel	6/3	ES/panel
MGH–USA					29	ES
LUMC–Netherlands					6	GS
**Total**	816		410		878	

*NCI* National Cancer Institute, *OUH* Oslo University Hospital, *UNIGE* Università degli Studi di Genova, *QIMR* Queensland Institute of Medical Research, *Sanger *Wellcome Sanger Institute, *KI* Karolinska Institute, *H. Bichat* Hôpital Bichat-Claude Bernard, *MGH* Massachussets General Hospital, *LUMC* Leiden University Medical Center.

^a^See Supplementary material for a complete list of the 22 contributing sites from the ten participant groups.

Where available, we collected similar information on 1,446 controls (individuals without a current or prior cancer diagnosis) who underwent *ATM* genotyping via ES (598) or panel sequencing (848). However, since controls were available for only 2 of 22 centers, we used the Genome Aggregation Database (gnomAD) [12] as the primary control group.

gnomAD version 2.1 consists of 125,748 exomes and 15,708 genomes from 141,456 germline DNA samples belonging to individuals enrolled in disease-oriented and population case–control studies. Since our study cohort included cases enrolled in countries with predominantly non-Finnish European (NFE) ancestry, we only used the gnomAD NFE subset, consisting of 64,603 individuals (56,885 exomes and 7,718 genomes). Moreover, we performed a secondary analysis comparing cases and controls from the two centers that provided controls.

### Variant selection and classification

All nonsense, frameshift, splice acceptor, and splice donor *ATM* variants found in either our study cohort or the NFE gnomAD cohort were considered LOF and included in the analyses (ATM RefSeq NM000051.4, LRG_135 and NP000042.3).

**Fig. 1:**
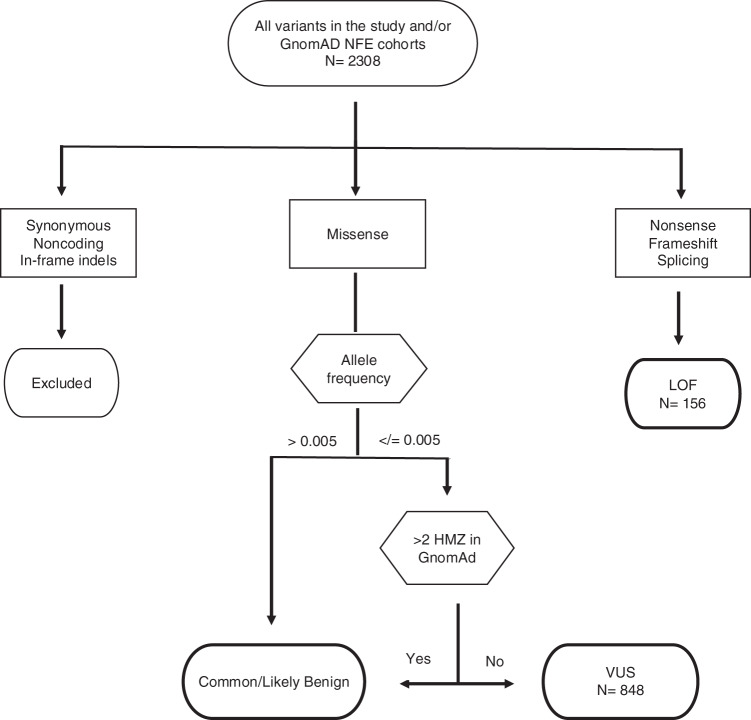
*ATM* variants selection criteria. HMZ individuals homozygous for an ATM variant, LOF loss of function, NFE non-Finnish European, VUS variant of uncertain significance.

The inclusion of missense variants was based on frequency criteria. Given that AT prevalence worldwide ranges from 1:40,000 to 1:100,000 [13], the estimated allele frequency of AT heterozygotes is 0.003–0.005. Therefore, all missense variants with an allele frequency (AF) above 0.005 were considered to likely be benign and were thus excluded from the analyses. Moreover, missense variants reported to be homozygous in more than two gnomAD subjects—and thus considered benign—were also excluded. All remaining missense variants were considered VUS and included in the further analyses (Fig. 1). In addition, we reviewed PubMed indexed articles to assess each of the LOF and VUS variants found in our case cohort to evaluate their relationships to AT or cancer.

### Statistical analysis and data visualization

For the purposes of our analysis, we grouped all LOF variants together and compared cases to NFE gnomAD controls. A similar approach was used for evaluating VUS. In addition, we directly examined individual variants if observed in ≥3 cases.

We compared the grouped LOF variants or VUS and individual variants (in ≥3 cases) in our cohort and NFE gnomAD data using Fisher’s exact tests. We also conducted analyses restricting the case sample to those considered to be genetically enriched: probands from melanoma-prone families and MPM cases. For VUS only, we repeated the analysis including variants depending on whether they were located in one of the three ATM functional domains, i.e., FRAP–ATM–TRRAP (FAT), phosphatidylinositol 3-kinase/phosphatidylinositol 4-kinase (PI3K/PI4K), and FAT carboxy-terminal (FATC), which encompass residues 1940–2566, 2712–2962, and 3024–3056, respectively.

In addition, we also compared grouped LOF variants or VUS in cases and controls from the two centers that provided controls.

All analyses were two-sided and a 0.05 cutoff was used for statistical significance. Statistical analyses were performed within the R computational environment [14].

The lollipop plot was generated using cBioportal Mutation Mapper [15, 16] and Adobe Illustrator® software.

## RESULTS

### Distribution of *ATM* variants in the study cohort compared to gnomAD

Our study cohort consisted of 2,104 cases (816 sporadic from case–control cohorts, 410 sporadic MPM, and 878 probands of melanoma-prone families). After filtering (see “Materials and Methods”), we retained 1,004 unique *ATM* variants, 156 LOF (12 from the study sample, 138 in NFE gnomAD, and 6 in both), and 848 unique VUS (42 in the study cohort, 731 in NFE gnomAD, and 75 in both).

**Fig. 2:**
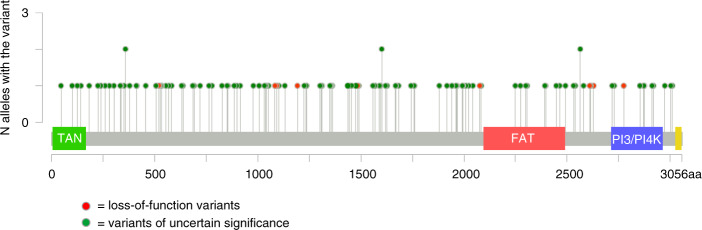
The lollipop plot shows the distribution of ATM loss-of-function (LOF) variants and variants of uncertain significance (VUS) found in the study cohort. FAT FRAP–ATM–TRRAP domain, PI3/PI4K phosphatidylinositol 3-kinase/phosphatidylinositol 4-kinase-related kinase domain, TAN Tel1/ATM N-terminal motif domain.

Our study cohort had 20 LOF alleles in 11 familial, 3 MPM, and 6 sporadic single primary melanoma (SPM) cases (0.95%, or 1.08% in the genetically enriched subset of familial + MPM cases). Moreover, there were 192 VUS alleles in 102 familial, 20 sporadic MPM, and 51 sporadic SPM cases. The distribution of LOF variants and VUS found in our study cohort across the ATM gene is shown in Fig. 2.

The NFE subset of the gnomAD cohort consists of 64,603 individuals, for a total allele count of 129,206. We observed 4,468 alleles with an *ATM* variant, including 223 LOF variants and 4,245 VUS.

We observed a higher frequency of LOF variants in the study cohort compared to the NFE gnomAD controls (AF 0.005 and 0.002, OR = 2.6, 95% CI = 1.56-4.11, *p* = 2.2e-04), with a slightly larger difference when we restricted the analysis to the genetically enriched subset (AF 0.0054 and 0.002, OR = 2.97, 95% CI = 1.6-5.09, *p* = 4.9e-04) (Table 2a).

**Table 2:** Frequency of *ATM* LOF/VUS variants in the study cohort and in the gnomAD database.

	Study cohort		gnomAD NFE		OR^a^ (95% CI)	*p* value
	*N* variant alleles/total alleles	AF	*N* variant alleles/total alleles	AF		
**a. LOF**						
All	20/4,208	0.005	237/129,206	0.002	2.6 (1.56–4.11)	2.2e-04
Fam + MPM	14/2,576	0.0054			﻿2.97 (1.6-5.09)	4.9e-04
**b. VUS**						
All	192/4,208	0.046	4,268/129,206	0.033	1.41 (1.21–1.64)	1.03E-02
Fam + MPM	135/2,576	0.053			1.63 (1.36–1.94)	3.06E-04

*AF* allele frequency, *CI*  confidence interval, *LOF* loss-of-function, *NFE* non-Finnish European, *OR* odds ratio, *VUS* variants of uncertain significance.

^a^Odds of finding the variants in the study cohort compared to the odds of finding the variants in the gnomAD NFE cohort.

One LOF variant, c.3576G>A, was observed in three unrelated Italian melanoma cases, at a frequency higher than in gnomAD NFE (AF = 0.0007 and 0.00004, OR = 20.25, 95% CI = 2.96–119.31, p = 0.001433). This variant, previously thought to be a synonymous variant p.(Lys1192=), is actually a splice variant p.(Ser1135_Lys1195del58), resulting in the skipping of the entire exon 26 of the *ATM* gene [17].

Evaluation of the VUS showed a similar, albeit smaller, association, both when considering all cases (AF 0.046 and 0.033, OR = 1.41, 95% CI = 1.21–1.64, *p* = 1.033e-05) and the familial/MPM subset (AF 0.053 and 0.033, OR = 1.63, 95% CI = 1.36–1.94, *p* = 3.059e-07) (Table 2b).

The association of VUS with melanoma was also present when the analysis was limited to variants located in one of the three *ATM* functional domains, in all cases (AF 0.013 and 0.009, OR = 1.36, 95% CI = 1.01–1.79, *p* = 0.0347) and in familial/MPM cases (AF 0.014 and 0.009, OR = 1.55, 95% CI = 1.08–2.15, *p* = 0.0129). Similar results were obtained for the subset of variants outside the *ATM* functional domains: AF 0.033 and 0.024, OR = 1.41, CI = 1.17–1.67, *p* = 2.205e-04 (all cases); AF 0.038 and 0.024, OR = 1.63, CI = 1.31–2, *p* = 1.19e-05 (familial/MPM cases).

Thirteen VUS, found in three or more cases of our study cohort, were evaluated individually (Table S1). Variant c.1368A>C p.(Leu456Phe) was only found in our study cohort. For ten variants, the allele frequency was higher in our study cohort than in gnomAD; for one of these variants, c.5750G>C p.(Arg1917Thr), the difference was statistically significant (*p* < 0.05). Although, to our knowledge, there are no other studies associating c.5750G>C with melanoma, this variant has been described in cancer patients. The c.5750G>C variant was found in familial cases with hereditary breast and ovarian cancer enrolled in the GENESIS study cohort [18]. Two of the other VUS, c.1229T>C p.(Val410Ala) and c.6067G>A p.(Gly2023Arg), have been reported in AT patients .[19, 20] Moreover, c.1744T>C p.(Phe582Leu), c.1229T>C, and c.6919C>T p.(Leu2307Phe) have also been previously described in individuals with hematologic malignancies [21, 22].

Of the two VUS with AF more frequent in controls, albeit not significantly (*p* > 0.05), c.998C>T p.(Ser333Phe) was previously found in a child with acute leukemia who was homozygous for this variant, but without an AT diagnosis, and in a nonsyndromic homozygous colorectal cancer patient, which suggests that this variant may be benign [22, 23]. The other variant that was more frequent in controls than cases, c.3925G>A p.(Ala1309Thr), was found in 1/7,051 breast cancer cases of a Japanese case–control study cohort [24].

Additional information on all variants found in our study cohort, including previously published reports in patients with AT or cancer, as well as predictions of pathogenicity using in silico tools, is shown in Table S3.

### Distribution of *ATM* variants in cases and controls from two centers

There were 1,142 cases from the Genoa and National Cancer Institute (NCI) centers, consisting of 396 familial melanoma probands and MPM cases, plus 746 sporadic melanoma cases. The control groups, recruited by the same centers, consisted of 1,446 individuals without a current or prior history of melanoma. LOF variants and VUS found in controls are shown in Table S4.

We found *ATM* LOF variants in 12 cases and 6 controls (AF 0.005 and 0.002, respectively, OR = 2.54, 95% CI = 0.88–8.26, *p* = 0.06). The difference was more pronounced and significant when we restricted the analysis to the genetically enriched subset (i.e., high-risk melanoma cases), namely familial and MPM cases (AF 0.008 and 0.002, OR = 3.67, 95% CI = 0.98–13.77, *p* = 0.027).

Overall, VUS appeared to be more frequent in controls (AF = 0.039) than in cases (AF = 0.023, OR = 0.56, 95% CI = 0.40–0.80, *p* = 0.0008); however, when the analysis was restricted to high-risk cases, the results were consistent with those of LOF variants (AF 0.060 in cases and 0.040 in controls, OR = 1.54, 95% CI = 1.06–2.20, *p* = 0.018).

### Cosegregation of *ATM* variants and melanoma

*ATM* genotyping for affected family members with LOF variants was only available for six probands (Table 3). Of these, one family with two sequenced cases (UNIGE_47, c.3576G>A; p.Ser1135_Lys1195del58) and one family with three sequenced cases showed complete cosegregation of the variant (c.1236dup; p.Leu413Alafs*17) with melanoma, whereas a third family showed partial cosegregation (c.7829_7830del; p.Arg2610Lysfs*2, 2/3 cases with the variant). Similarly, family Sanger_7 showed partial cosegregation, as three of four sequenced first-degree relatives shared the same ATM variant (c.1561_1562delAG; p.Glu522Ilefs*43). Interestingly, although the proband had only the ATM frameshift variant, two other siblings had a concurrent *CDKN2A* LOF variant, whereas a fourth sibling only had a *CDKN2A* variant. Regardless of which of the two genes was altered at the germline level, all four individuals developed melanoma. The parents of the proband, both untested, were diagnosed with melanoma (father) and breast cancer (mother).

**Table 3:** Melanoma cases with *ATM* LOF variants in the study cohort.

ID	Selection criteria	Nucleotide base change	Amino acid change	Variant effect	Cases	Cosegregation	Total number of melanomas	Age at diagnosis of first melanoma	Personal history of other cancers	Family history of other cancers
NCI_1	S	c.717_720del	p.Phe239Leufs*15	FS	1	n.d.	1	n.a	n.a	n.a
NCI_2	S	c.6228delT	p.Leu2077Phefs*5	FS	1	n.d.	1	n.a	n.a	n.a
NCI_3	S	c.7928-2A>T		Splice	1	n.d.	1	n.a	n.a	n.a
NCI_4	S	c.7629 + 2T>C		Splice	1	n.d.	1	n.a	n.a	n.a
NCI_5	S	c.902-1G>T		Splice	1	n.d.	1	n.a	n.a	n.a
UNIGE_4	F	c.3576G>A	p.Ser1135_Lys1195del58	Splice	3	n.d.	2	41	NO	NO
UNIGE_22	F	c.3576G>A	p.Ser1135_Lys1195del58	Splice	2	n.d.	1	49	NO	NO
UNIGE_47	F	c.3576G>A	p.Ser1135_Lys1195del58	Splice	2	Yes (2/2)	2	40	BCC	NO
UNIGE_24	F	c.4451delT	p.Met1484Argfs*15	FS	3	n.d.	3	47	HL, PC	CRC, PC
UNIGE_37	F	c.8319_8323dupTGTCC	p.Pro2775Leufs*33	FS	2	n.d.	3	48	BCC, BC	PrC, BCC
UNIGE_39	MPM	c.5979_5983delTAAAG	p.Ser1993Argfs*23	FS	1	n.d.	3 (1CMM, 2UM)	51	NO	NO
UNIGE_40	S	c.3275C>A	p.Ser1092*	NS	1	n.d.	1	47	NO	n.a
QIMR_7	F	c.1236dup	p.Leu413Alafs*17	FS	3	Yes (3/3)	n.a	59	NO	NO
QIMR_1	F	c.7886_7890del	p.Ile2629Serfs*25	FS	8	No (1/6)	1	25	NO	CRC
QIMR_15	F	c.7829_7830del	p.Arg2610Lysfs*2	FS	3	Partial (2/3)	12	40	EC	CRC, MES
QIMR_16	F	c.1236-3_1236-2delinsATTT		FS	7	No (1/3)	n.a	n.a	n.a	n.a
OUH_8	MPM	c.3244_3245insTG	p.His1082Leufs*28	FS	1	n.d.	3	72	NO	n.a
OUH_13	MPM	c.3284 + 1G>A		Splice	1	n.d.	3	28	NO	n.a
Sanger_7	F	c.1561_1562delAG	p.Glu522Ilefs*43	FS	5	Partial (3/4)	2	22	n.a	BC
Bichat_3	F	c.8850 + 2dup		Splice	3	n.d.	3	51	CLL	n.a

Cosegregation = *N* cases with a LOF variant/*N* sequenced cases.

*BC*  breast cancer, *BCC*  basal cell cancer, *CLL*  chronic lymphocytic leukemia, *CMM * cutaneous malignant melanoma, *CRC  *colorectal cancer, *EC * endometrial cancer, *F*  familial melanoma, *FS*  frameshift variant, *HL*  Hodgkin lymphoma, *LOF*  loss of function, *MES*  mesothelioma, *MPM*  multiple primary melanoma, *n.a.* not available, *n.d.* not determined, *NS*  nonsense variant, *PC*  pancreatic cancer, *PrC* prostate cancer, *S* sporadic, *Splice* splice acceptor or splice donor variant, *UM*  uveal melanoma.

Conversely, in one large family, only one of six affected members sequenced had an *ATM* LOF variant (c.7886_7890del; p.Ile2629Serfs*25). However, as the affected mother of patient QIMR_1 did not carry the c.7886_7890del, this variant was either a de novo germline variant or was inherited from the unaffected father for whom no information on family history was available and, therefore, cosegregation could not be determined. Family QIMR_15 showed no evidence of cosegregation, as only one of three analyzed affected family members had the c.7829_7830del p.(Arg2610Lysfs*2) variant; however, no information was available on four additional family members who had melanoma but were not genotyped for *ATM*.

Information on cosegregation was available for 40 of 101 familial melanoma probands with VUS. Of these, 29 families with a single *ATM* variant showed evidence of cosegregation with melanoma in sequenced affected family members, and 9 probands showed partial cosegregation in their families. Among cases with two *ATM* variants, both variants were present in sequenced family members of NCI-Mel_2 and NCI-Mel_16 MelaNostrum families. (Table S2). Overall, most families included only two melanoma cases that were sequenced. Only 6 of 13 families with at least 3 melanoma cases sequenced showed full cosegregation.

### *ATM* variants and nonmelanoma cancers

Nonmelanoma tumors were reported in a subset of cases and/or their families. Of the 20 probands with LOF variants, information on nonmelanoma tumors was available on 14 probands. Of these, six were diagnosed with a nonmelanoma cancer, and four of these cases also had at least one first-degree relative with a nonmelanoma cancer. Namely, the c.8850 + 2insA splice variant was found in a melanoma patient who also developed chronic lymphocytic leukemia (CLL). This variant, absent in gnomAD, has not been previously reported in AT or cancer patients. Separately, the patient harboring the c.4451delT p.(Met1484Argfs*15) variant was diagnosed with Hodgkin lymphoma and pancreatic cancer and had a positive family history of pancreatic and colorectal cancer (CRC). Moreover, c.8319_8323dupTGTCC p.(Pro2775Leufs*32) was found in a woman who was diagnosed with basal cell carcinoma (BCC) and breast cancer at age 49 and 53, respectively, and who had a first-degree relative with prostate cancer and BCC. BCC also occurred in one of the three cases with the c.3576G>A p.(Ser1135_Lys1195del58). c.1561_1562delAG p.(Glu522Ilefs*43) was found in a familial melanoma patient of our cohort who developed a BCC and whose mother was diagnosed with breast cancer, as reported in the previous paragraph. This variant, as well as similar deletions that cause the same frameshift with a premature stop codon, resulting in absent ATM kinase activity, have been found in AT patients [25].

The patient harboring the c.7829_7830del p.(Arg2610Lysfs*2) variant was diagnosed with endometrial cancer and had a positive family history for colorectal cancer. The c.7886_7890del p.(Ile2629Serfs*25) variant was found in a patient with no other cancers other than melanoma but with a first-degree relative who developed CRC at age 61. This variant, which results in absent ATM protein expression [26], has been found in homozygous and compound heterozygous AT [26], and it has been associated with an increased breast cancer risk in heterozygotes [27]. Other *ATM* LOF variants were found in individuals without personal or family history of other cancers or for whom this information was not available. However, c.5979_5983delTAAAG p.(Ser1993Argfs*22), as well as c.6228delT p.(Leu2077Phefs*5), have been previously described in CLL and pancreatic cancer, respectively [28, 29]. Moreover, c.717_720del p.(Phe239Leufs*15) is a known *ATM* pathogenic variant found in AT patients [30]. To our knowledge, the remaining LOF variants have never been reported in the literature in relation to AT or other cancers.

Personal history of nonmelanoma cancers was reported in 32 of 110 cases with VUS for whom this information was available (29%). Among these, the most frequent was nonmelanoma skin cancer (NMSC), which was present in 9 cases (7 BCC, 1 squamous cell carcinoma, and 1 NMSC not otherwise specified), followed by prostate cancer (6 cases), breast cancer (6 cases), and lymphoma (3 cases). Kidney, bladder, endometrial, CRC, pancreatic, thyroid, lung cancer, as well as mesothelioma, leiomyosarcoma, meningioma, glioblastoma, and ovarian teratoma were each found in one patient.

Family history could be retrieved for 77 cases with VUS. Of these, 32 cases (41%) had at least one first-degree relative diagnosed with nonmelanoma cancers (Table S2). The most frequent cancer was pancreatic cancer, found in 8 families, followed by breast cancer (6 families), hematological malignancies (5 families), lung cancer, and prostate cancer (4 families each). For a complete overview of nonmelanoma cancers in our cohort, see Tables 3 and S2.

## DISCUSSION

Despite the technological advances that have occurred during the last decades that led to multiple discoveries in the study of the human genome and cancer, several questions are still partially unanswered. For instance, much of the heritability of melanoma cannot be explained by germline pathogenic variants in single established melanoma predisposition gene(s). In contrast, an increased prevalence of melanoma cases has been found in known familial cancer syndromes for which an association with melanoma was not previously established [4]. An example of this phenomenon is exemplified by *ATM*, an intermediate-risk breast cancer susceptibility gene that has been implicated in melanoma susceptibility [6, 9]. Considering that the risk of melanoma may be analogous to or even lower than that of breast cancer, collecting information on pedigrees large enough to investigate cancer predisposition is a challenge. An additional level of uncertainty is that different types of variants may confer divergent cancer risks, which can be hypothesized based on prior knowledge derived from studies on AT and high-risk breast cancer patients [31].

To our knowledge, there are no other hypothesis-driven studies investigating rare *ATM* variants in large international multicenter melanoma cohorts. Here, we demonstrated that *ATM* LOF variants are more frequent in melanoma patients than in NFE subjects in the gnomAD database, seemingly conferring moderate risk, supporting *ATM* as a melanoma predisposition gene. Overall, *ATM* LOF variants were observed in approximately 1% of the melanoma cases in our study cohort (0.95% in the whole cohort, 1.08% in FAM + MPM cases and 0.7% in sporadic cases), more than in gnomAD NFE samples (0.36%), and approximately less than that reported for the known moderate melanoma risk variant *MITF* p.Glu318Lys, which ranges between 1.8% and 3.6% across different studies [32–35]. This, together with the effect estimates observed in our study, suggests that *ATM* might have a similar burden and act as a moderate risk gene in melanoma, similar to what has been observed in breast cancer patients [36]. However, the frequency reported here was slightly higher than reported by recent large ES and GS studies. Namely, The Cancer Genome Atlas Consortium (TCGA) found germline *ATM* LOF variants in only 3 of 470 (0.6%) melanoma patients analyzed for a pan-cancer ES study [37]. However, in contrast to the current study sample, the TCGA study cohort was not selected based on family history of melanoma. Indeed, the majority of LOF variants in our cohort were found in high-risk melanoma patients, that is, either belonging to melanoma-prone families or diagnosed with MPM, whereas the rate of LOF variants in sporadic cases in our study is only slightly higher than that of the TCGA study cohort. Previous studies investigating ATM variants showed a higher prevalence of deleterious variants in familial and MPM cases (up to 3%) than that of LOF variants found here. However, those estimates included missense variants that were classified as deleterious/pathogenic [10, 11] according to in silico prediction tools, cosegregation, and literature data, while here missense variants were classified only as VUS based on a frequency criteria (see “Materials and Methods”).

The association between *ATM* VUS, especially those located in functional domains, and melanoma was weaker than that observed for LOF variants. One possible explanation is that, as for *ATM* itself, missense variants confer a lower risk compared to truncating (i.e., LOF) variants. However, although we only included rare VUS missense variants that were not found homozygous in healthy individuals, it is likely that nonpathogenic variants in this group might have diluted an association with melanoma. The fact that the subset of VUS in functional domains showed a smaller OR in our cohort compared to all VUS could also hint at the possibility that missense variants are less involved in melanoma predisposition than LOF variants. However, restricting the analysis to functional domains reduces the number of VUS and the number of individuals carrying them (both in our study cohort and in gnomAD), and therefore this difference could be simply due to the need of a larger sample size and/or to the exclusion of potentially functional variants outside the three functional domains, such as missense variants that alter protein folding. Indeed, the association with melanoma was maintained for VUS outside *ATM* functional domains, which may be consistent with this latter hypothesis. Thus, the reported association might have been stronger if selection based on functional assessment were possible for all missense/VUS variants.

In our cohort, personal and/or family history for *ATM*-related cancers, specifically breast cancer, pancreatic cancer, and hematologic malignancies, was found in 5/19 cases heterozygous for LOF variants (26%). Although information on other tumors was only available for a subset of them, the abovementioned tumors were found in 8/103 (7.7%) probands with VUS and in first-degree relatives of 20/78 (25,6%) probands with VUS. Since these individuals were recruited mainly based on their personal and family history of melanoma, it is therefore unlikely that an enrichment of families with ATM-related tumors may have biased our results.

Our study design presents some limitations. The lack of availability of healthy individuals from the majority of the study groups made it impossible to perform a case–control study with in-house controls, sequenced with the same platforms and similar coverage, and, therefore, we compared our study cases against population controls from a publicly available database (gnomAD). Aware of the risk of population stratification bias due to nonoverlapping distributions of ethnic groups, we used the gnomAD NFE cohort as our control group, since the affected individuals in our study cohort either come from European countries excluding Finland, or from American and Australian centers that are composed mainly of individuals of European descent. Even with this adjustment, the possibility of population stratification bias cannot be completely excluded. However, although underpowered, a case–control analysis limited to the centers that provided ethnically matched controls showed an enrichment of *ATM* LOF variants and VUS in high-risk melanoma cases, consistent with the results from the main analyses.

Another limitation is that, although gnomAD is composed of data sets from several studies, it includes cancer cohorts, and, therefore, the detection of pathogenic *ATM* variants in this data set could be due, at least in part, to the presence of affected individuals with *ATM*-induced germline cancer predisposition, such as breast or pancreatic cancer cases in the TCGA data set. Therefore, the association of *ATM* pathogenic variants with melanoma could be higher than the one we observed. Another issue is that the case groups differed by study design, sequencing platform, and country of origin. It is not clear how this heterogeneity might have influenced the results. However, given the relatively small individual sample sizes from each group, it was not feasible to analyze individual group data separately, and therefore we analyzed all contributed data together. We also conducted subset analyses on cases purported to have the highest underlying genetic risks (familial and MPM cases). As hypothesized, we observed the strongest association in this genetically enriched subset.

If confirmed by further studies, our results could provide benefits in the clinical setting. In the era of personalized medicine, DNA damage repair genes are promising targets for novel cancer therapies. Poly (ADP-ribose) polymerase (PARP) inhibitors, for example, are used for *BRCA1*/2-positive breast, ovarian, and pancreatic cancer [38, 39] and have been FDA approved in the United States for castration-resistant *ATM*-deficient prostate cancer following a recent clinical trial [40]. Recent studies have also shown their potential role in the treatment of other *ATM*-deficient cancers [6], and clinical trials on patients with potentially actionable ATM-deficient cancers are ongoing.

The implication of *ATM* in melanoma development is recent, but the activation of the ATM/ATR pathway in response to UV-induced replication stress has been documented [7]. Thus, it is possible a defective activation of this pathway leads to malignant transformation and, if this is the case, *ATM* penetrance could be modulated by UV exposure and/or the co-occurrence of other inherited melanoma predisposing factors, such as phototype and *MC1R* variants.

This study is part of a broader project aimed at fully exploring the link between *ATM* and melanoma. Indeed, in addition to LOF variants, rare missense variants were enriched in our melanoma cohort. To gain a clearer picture of the impact of *ATM* on melanoma, it will be necessary to carry out pathogenicity assessment of rare missense variants through functional testing. Besides its nuclear role in double-strand breaks and cell cycle checkpoint, cytoplasmic ATM plays noncanonical roles in the regulation of organelle/oxidative/energetic metabolism, which may involve melanoma biogenesis, and potentially, melanoma treatment [41, 42].

Moreover, an assessment of the magnitude of ATM risk in melanoma will be crucial to determine the potential clinical utility of germline *ATM* testing in terms of surveillance. In summary, the findings from this study support the designation of *ATM* as a moderate-risk melanoma susceptibility gene.

## Supplementary information

Supplementary Methods

Supplementary tableS2

Supplementary tableS3

Supplementary tableS4

## Data Availability

The data that support the findings of this study are available from the corresponding author upon request.
