# Supplementary material for: Germline ATM variants predispose to melanoma: a joint analysis across the GenoMEL and MelaNostrum consortia
Source: Genet Med. 2021 Jul 14;23(11):2087–95. doi: 10.1038/s41436-021-01240-8 (PMC8553617; doi:10.1038/s41436-021-01240-8)
Supplement: Supplementary file 1 — Supplementary Methods [file 41436_2021_1240_MOESM1_ESM.pdf]

SUPPLEMENTARY METHODS

A total of 22 sites from 10 partecipating centers belonging to either the GenoMEL Consortium, the Melanostrum consortium or both, collected their ATM study cohort and DNA sequencing data (Table 1). Of these, three centers (Melanostrum-NCI, Sanger institute and QIMR Berghofer) performed also sequencing and data collection for other sites. All sites, as part of the GenoMEL and Melanostrum consortium, used shared selection criteria for MPM and familial melanoma cases[1], described in

specific references for each site below, and/or previously developed in the consortium published common recommendations (www.genomel.org). See below for a detailed description of the study cohort.

List of participating sites: NCI, Oslo, Genoa, QIMR Berghofer (QIMR, Copenhagen, Sydney, Stockholm, Barcelona), Melanostrum_NCI (Cesena, Genoa, Milan, Padua, L'Aquila, Rome, Valencia, Barcelona.), Wellcome Trust Sanger Institute (Stockholm, Leiden, Leeds, London, Copenhagen, Sydney, Barcelona, Philadelphia), Stockholm, Paris, Boston, Leiden.

GENOMEL CONSORTIUM

DIVISION OF CANCER EPIDEMIOLOGY AND GENETICS, NATIONAL CANCER INSTITUTE (NCI), BETHESDA, USA

Description of the study cohort

The CMM cases for this study were part of a family study that has been previously described[2]. All study participants were of European ancestry. For this study, *ATM* data from WES were available for 72 familial melanoma probands and 14 single high-risk melanoma patients with early age at diagnosis (n=11) or multiple primary melanomas (n=3). Data on *ATM* from 598 population controls from two cohort studies (Cancer Prevention Study

[CPS]-II, n = 223; PLCO, n = 375)[2,3] were available from WES for inclusion in the current study to evaluate genetic burden for *ATM*. SeqCAP EZ Human Exome Library v3.0 or v3.0+UTR (Roche NimbleGen, Madison, WI) was utilized for exome sequence capture. The captured DNA was then subject to paired-end sequencing utilizing the Illumina HiSeq2000 sequencer for 2 X 100-bp sequencing of paired-ends (Illumina, San Diego, CA). Exome sequencing was performed to a sufficient depth to achieve a minimum coverage of 15 reads in at least 80% of the coding sequence from the UCSC hg19 transcripts database. The sequencing/analysis methods for the cases and population control samples followed a similar ensemble calling process. Variant calling for the population controls was done together with that for an in-house database of cancer-prone families that included the melanoma families.

A targeted sequencing panel was used to sequence the entire exonic region for *ATM* in 783 CMM cases and 768 controls from the PLCO and Agriculture Health Study (AHS) studies. Deep sequence coverage was generated for each sample. Genotypes were determined independently for each sample based on the ratio of base calls in those sequence reads at a given locus.
WES and targeted sequencing for ATM was performed at the Cancer Genomics Research Laboratory, Division of Cancer Epidemiology and Genetics, National Cancer Institute (CGR, DCEG ,NCI), as previously described[2,3].
Ethics Statement. All family members who were willing to participate in the study provided written informed consent under an NCI IRB approved protocol.

OSLO UNIVERSITY HOSPITAL (OUH), OSLO, NORWAY

The Norwegian cohort (N= 279) was selected among 390 MPM patients previously tested for *CDKN2A*/*CDK4* germline pathogenic variants[4]. In short, patients being 18 years or older, and diagnosed with ≥ 2 melanomas of which one could be *in situ*, were recruited through the Cancer Registry of Norway (CRN). The 279 patients were all confirmed *CDKN2A*/*CDK4* negative and priority was given to patients with, 1) ≥ 3 malignant melanomas (16.1%), 2) ≥ 3 melanomas including in situ (47.3 %), and 3) additional non-melanoma cancers (36%). A family history was reported in one of five (20.8 %) of the patients included.

Ethical statement: Data of patients included in this study were de-identified, and access to updated clinical data was granted from CRN in February 2016 in accordance with approval by the Regional Ethical Committee.

UNIVERSITY OF GENOA (UNIGE), GENOA, ITALY

Description of the study cohort: 273 melanoma cases and 80 cancer-free controls from Genoa, as well as DNA extraction and gene panel sequencing methods have been previously described[5]. Additional melanoma cases from Genoa were included and sequenced as part of the MelaNostrum consortium (see below).

Ethics Statement. All subjects enrolled in this study signed an informed consent for genetic testing and genetic-based research under local Institutional Review Board (IRB) approved protocols (CE AOU San Martino Genova 10/2010).

QIMR BERGHOFER MEDICAL RESEARCH INSTITUTE, BRISBANE ,AUSTRALIA

Description of the study cohort. The Queensland cohort comprised of participants from melanoma families recruited to the Queensland Familial Melanoma Project (QFMP) and The Queensland Study of Melanoma: environmental and genetic associations (Q-MEGA), together with participants recruited following referral from specialist clinicians. Cases and their families provided blood/saliva samples from which DNA was extracted[6,7]. QIMR Berghofer also collected melanoma samples from other GenoMel centers, i.e. Denmark, Sweden, Sydney.

DNA was extracted from saliva and blood samples using standard protocols, sequenced by Macrogen (South Korea), and data were aligned and analyzed as described elsewhere[8].

Ethics Statement. Participants were consented for this study under ethics approval granted by the QIMR Berghofer Medical Research Institute Human Research Ethics Committee (HREC, reference number: HREC/14/QPAH/495), and partecipants' data were de-identified.

WELLCOME TRUST SANGER INSTITUTE, CAMBRIDGE, UK

Wellcome Trust Sanger Institute performed WES and WGS for collected germline DNA samples collected from Sweden (Karolinska Institute), Leiden, Leeds, London, Copenhagen, Sydney, Barcelona, University of Pennsylvania.

Whole-genome sequencing was performed using the Illumina HiSeq 2500/HiSeqX platform with 150bp paired-end reads, 200bp to 1000bp insert size and average read depth of 35x. For the WES, exonic regions were captured with the Agilent SureSelect Target Enrichment System. Paired-end reads of 75bp were generated on Illumina the HiSeq2000 platform. A subset of samples were sequenced at the Beijing Genomics Institute using the Illumina HiSeq2000 platform with 90 bp paired-end reads. Sequencing reads were aligned to the GRCh38 reference genome with BWA-mem version 0.7.15-r1140. Raw variants were called using GATK's HaplotypeCaller (v. 3.7-0-gcfedb67) resulting in a call set in a multi-sample VCF file format. Data of all patients whose DNA was sequenced by Wellcome Trust Sanger Institute were de-identified.

KAROLINSKA INSTITUTE, STOCKHOLM, SWEDEN

Description of the study cohort. Melanoma patients have previously been recruited when visited the Oncology Clinic at the Karolinska University Hospital. Melanoma families were defined as kindreds with two first-degree relatives with melanoma or three or more melanomas in the family. Gene panel sequencing of 10 multiple primary melanoma patients and 78 familial melanoma patients (from 59 families) was executed using the HaloPlex Target Enrichment kit (fast protocol), followed by sequencing on a HiSeq 2000 instrument (Illumina, San Diego, CA). The procedure is described in Tuominen et.al Genes, Chromosomes & Cancer 55:601–611 (2016). Twenty-four patients belonging to 14 different melanoma families was analyzed by whole exome sequencing applying the TrueSeq Exome Enrichment Kit and sequencing using Illumina HiSeq 2000 (Illumina). Both panel sequencing and WES was done as a service at the Science for Life laboratory in Stockholm.

Ethics Statement. Data of patients recruited for this study were de-identidied. The research was approved by the KI research ethical committee north and the Stockholm research ethical review board.

HÔPITAL BICHAT, PARIS, FRANCE

Description of the study cohort. This cohort comprised 31 sporadic multiple primary melanoma cases, as well as 9 probands belonging to melanoma-prone families. *ATM* germline mutational status was obtained through WES in 20 multiple primary melanoma and 6 familial melanoma cases and by panel sequencing in the remaining melanoma cases.

Ethics Statement. Written informed consent was obtained from all participant, and individual data were de-identified.

MASSACHUSETTS GENERAL HOSPITAL, BOSTON, USA

Description of the study cohort. The study cohorts from Boston, Massachusetts, USA and Athens, Greece, have been described in a prior manuscript[9].

Ethics Statement. All patients provided written informed consent prior to participation. The study protocol was approved by the Scientific and Ethics Committee of the Massachusetts General Hospital in Boston, Massachusetts, USA and the Andreas Sygros Hospital in Athens, Greece.

LEIDEN UNIVERSITY MEDICAL CENTER (LUMC), LEIDEN, THE NETHERLANDS

Description of the study cohort. Blood DNA samples were collected from 27 cutaneous melanoma patients of 6 different Dutch families with at least 4 affected family members. DNA sequencing was performed at Wellcome Trust Sanger Institute (see above).

Ethics Statement. Melanoma families were recruited at LUMC, Leiden, the Netherlands, with written informed consent of all participants (ethics approval number, P00.117).

UNIVERSITY HOSPITAL OF COPENHAGEN, DENMARK

Description of the study cohort. 54 families with 3 or more affected family members, from this study were selected were selected for this study from a previously described melanoma cohort [10].

WGS or WES was performed on blood samples either at QIMR Berghofer Medical Research Institute or at Wellcome Trust Sanger Institute (see above).

Ethics Statement. All participants signed informed consent prior to enrollment in the study. The Project was approved by the Ethics committee of the capital region of Copenhagen.

WESTMEAD INSTITUTE FOR MEDICAL RESEARCH, UNIVERSITY OF SYDNEY, AUSTRALIA

Consented participants and their family members were recruited between 1990-2019 as part of the multi-case melanoma family study. Melanoma and other cancer history were confirmed from pathology and medical records. Germline DNA sequencing was performed at Wellcome Trust Sanger Institute by WES and WGS, or at QIMR Berghofer Medical Research Institute by WES (see above).

Ethics statement. All participants signed informed consent prior to recruitment to the study. This study was approved by the NSW Population and Health Services Human Research Ethics Committee HREC/13/CIPHS/71, AU.

UNIVERSITY OF PENNSYLVANIA, PHILADELPHIA, USA

Description of the study cohort: Probands with melanoma or at increased risk for melanoma with a reported family history of disease were identified at the Pigmented Lesion Clinic of the University of Pennsylvania. Probands and their family members including individuals with and without melanoma were recruited between 1999-2011 as previously described[11]. Germline DNA sequencing was performed either by WES or WGS at Wellcome Trust Sanger Institute (see above).

Ethics statement. Data of patients included in this study were de-identified. This study was approved by the Institutional Review Board of the University of Pennsylvania (Protocol #700347).

MELANOSTRUM CONSORTIUM

Description of the study cohort. The following MelaNostrum consortium centres provided germline ATM sequencing data for this study: Cesena, Milan, Padua, L'Aquila, Rome, Genoa, Valencia, Barcelona. For each center, recruitment of cases and DNA sample collection has been previously described[12,13].

Whole exome sequencing on germline DNA samples and bioinformatic analysis has been performed at Division of Cancer Epidemiology and Genetics, National Cancer Institute, National Institutes of Health, Bethesda, MD, USA, as described in previous publications[13].

Ethics Statement. Recruitment of melanoma cases was carried out upon approval by local ethics committee and written informed consent was signed by all participants as described in Landi et al. [12,13].

REFERENCES

1. Leachman SA, Lucero OM, Sampson JE, et al. Identification, genetic testing, and management of hereditary melanoma. *Cancer Metastasis Rev*. 2017;36(1):77-90. doi:10.1007/s10555-017-9661-5
2. Goldstein AM, Xiao Y, Sampson J, et al. Rare germline variants in known melanoma susceptibility genes in familial melanoma. *Hum Mol Genet*. 2017;26(24):4886-4895. doi:10.1093/hmg/ddx368
3. Yang XR, Rotunno M, Xiao Y, et al. Multiple rare variants in high-risk pancreatic cancer-related genes may increase risk for pancreatic cancer in a subset of patients with and without germline CDKN2A mutations. *Hum Genet*. 2016;135(11):1241-1249. doi:10.1007/s00439-

016-1715-1

4. Helsing P, Nymoen DA, Ariansen S, et al. Population-based prevalence ofCDKN2A andCDK4 mutations in patients with multiple primary melanomas. *Genes, Chromosom Cancer*. 2008;47(2):175-184. doi:10.1002/gcc.20518
5. Pastorino L, Andreotti V, Dalmasso B, et al. Insights into Genetic Susceptibility to Melanoma by Gene Panel Testing: Potential Pathogenic Variants in ACD, ATM, BAP1, and POT1. *Cancers (Basel)*. 2020;12(4):1007. doi:10.3390/cancers12041007
6. Aitken JF, Green AC, MacLennan R, Youl P, Martin NG. The Queensland familial melanoma project: Study design and characteristics of participants. *Melanoma Res*. 1996;6(2):155-165. doi:10.1097/00008390-199604000-00011
7. Baxter AJ, Hughes MC, Kvaskoff M, et al. The Queensland Study of Melanoma: Environmental and Genetic Associations (Q-MEGA); study design, baseline characteristics, and repeatability of phenotype and sun exposure measures. *Twin Res Hum Genet*. 2008;11(2):183-196. doi:10.1375/twin.11.2.183
8. Pritchard AL, Johansson PA, Nathan V, et al. Germline mutations in candidate predisposition genes in individuals with cutaneous melanoma and at least two independent additional primary cancers. Haass NK, ed. *PLoS One*. 2018;13(4):e0194098. doi:10.1371/journal.pone.0194098
9. Artomov M, Stratigos AJ, Kim I, et al. Rare Variant, Gene-Based Association Study of Hereditary Melanoma Using Whole-Exome Sequencing. *JNCI J Natl Cancer Inst*. 2017;109(12). doi:10.1093/jnci/djx083
10. Wadt KAW, Aoude LG, Krogh L, et al. Molecular characterization of melanoma cases in Denmark suspected of genetic predisposition. *PLoS One*. 2015;10(3):e0122662. doi:10.1371/journal.pone.0122662
11. Taylor NJ, Mitra N, Goldstein AM, et al. Germline Variation at CDKN2A and Associations with Nevus Phenotypes among Members of Melanoma Families. *J Invest Dermatol*. 2017;137(12):2606-2612. doi:10.1016/j.jid.2017.07.829
12. Landi MT, Bishop DT, MacGregor S, et al. Genome-wide association meta-analyses combining multiple risk phenotypes provide insights into the genetic architecture of cutaneous melanoma susceptibility. *Nat Genet*. 2020;52(5):494-504. doi:10.1038/s41588-020-0611-8
13. Shi J, Yang XR, Ballew B, et al. Rare missense variants in POT1 predispose to familial cutaneous malignant melanoma. *Nat Genet*. 2014;46(5):482-486. doi:10.1038/ng.2941

Table S1. Frequency of single *ATM* VUS in the study cohort and in the gnomAD database

Nucleotide and Protein base change	Study cohort			GnomAD NFE			OR[a]	Lower CI	Upper CI	p-value
	N Alleles with the variant	Total N alleles	AF	N Alleles with the variant	Total N alleles	AF				
c.1229T>C ,(p.Val410Ala)	17	4210	0.00404	417	128912	0.00323	1.25	0.72	2.03	0.34
c.6067G>A ,(p.Gly2023Arg)	12	4210	0.00285	297	129142	0.0023	1.24	0.63	2.2	0.42
c.1744T>C ,(p.Phe582Leu)	9	4210	0.00214	138	128884	0.00107	2	0.89	3.91	0.05
c.544G>C ,(p.Val182Leu)	7	4210	0.00166	105	127084	0.00083	2.01	0.79	4.3	0.10
c.4424A>G ,(p.Tyr1475Cys)	6	4210	0.00143	117	128590	0.00091	1.57	0.56	3.52	0.29
c.998C>T ,(p.Ser333Phe)	5	4210	0.00119	212	128984	0.00164	0.72	0.23	1.71	0.69
c.3925G>A ,(p.Ala1309Thr)	4	4210	0.00095	156	129002	0.00121	0.79	0.21	2.05	0.82
c.4324T>C ,(p.Tyr1442His)	4	4210	0.00095	78	128820	0.00061	1.57	0.42	4.19	0.33
c.1368A>C ,(p.Leu456Phe)	3	4210	0.00071	-	-	-	-	-	-	-
c.5750G>C ,(p.Arg1917Thr)	3	4210	0.00071	2	113534	0.00002	40.51	4.64	481.19	0.00043
c.5975A>C ,(p.Lys1992Thr)	3	4210	0.00071	29	129010	0.00022	3.17	0.62	10.24	0.08
c.6919C>T ,(p.Leu2307Phe)	3	4210	0.00071	35	129078	0.00027	2.63	0.52	8.35	0.12
c.7187C>G ,(p.Thr2396Ser)	3	4210	0.00071	36	129086	0.00028	2.56	0.5	8.1	0.12

NFE= non-Finnish European, OR= odds ratio, CI= confidence interval. [a]Odds of finding the variant in the study cohort compared to the odds of finding the variant in the gnomAD NFE cohort
